# Use of Calcium Silicate-Based Materials in the Management of Open Apices: A Case Report

**DOI:** 10.7759/cureus.86471

**Published:** 2025-06-21

**Authors:** Bouchra Doumari, Sara Dhoum, Zineb El Hajjoui, Mouna Jabri

**Affiliations:** 1 Department of Conservative Dentistry and Endodontics, Faculty of Dentistry, Hassan II University, Casablanca, MAR

**Keywords:** apexification, calcium silicate, dental trauma, mineral trioxide aggregate, mta apexification, open apex management

## Abstract

Trauma in immature teeth can lead to open apices, posing challenges for treatment. Calcium hydroxide (Ca(OH)_2_), the traditional option, has limitations such as lengthy treatment times and risks of fracture. Mineral trioxide aggregate (MTA) has emerged as a superior alternative, offering excellent sealing and biocompatibility. This paper describes managing an immature central incisor in a 19-year-old male with necrotic pulp using an MTA apical plug, with a 24-month follow-up. The presented case outlines the difficulties encountered in treating an immature tooth with an acute apical abscess due to prior trauma. Challenges such as a wide apex, periradicular infection, and thin root walls complicated treatment decisions. Nevertheless, prioritizing the preservation of the tooth's function and aesthetics, the decision was made to utilize MTA apical plugs. MTA, known for its superior properties, including sealing ability, antibacterial efficacy, and biocompatibility, emerged as the treatment of choice. The case underscores the importance of MTA in endodontic practice, particularly in cases with open apices and periapical inflammation. Techniques such as proper MTA placement and adjunctive therapies like Ca(OH)_2_ dressing contribute to successful outcomes. Comparative studies highlight the effectiveness of MTA and Biodentine in apical sealing, with both materials demonstrating comparable performance. However, the technique used for apical plug formation influences microleakage, emphasizing the importance of precise placement methods. Overall, the case report contributes to the understanding of MTA's role in managing challenging endodontic cases, underscoring its efficacy in promoting periapical healing and preserving tooth structure and function. The MTA barrier as a treatment for apexification does not require multiple appointments, and the barrier formation does not need an external factor to develop, as in the case of Ca(OH)_2_ apexification, as well as in pulp regeneration.

## Introduction

In immature teeth, trauma can lead to pulp necrosis and cessation of tooth development, resulting in open apices. Treating non-vital permanent teeth with open apices poses challenges due to the large canal space, wide open apex, and thin, fragile root walls [1]. It is crucial to establish an efficient apical barrier to prevent apical leakage into the periradicular tissues and to ensure effective compaction of the root canal filling material without apical extrusion [2]. Calcium hydroxide (Ca(OH)_2_) is commonly used to create the apical calcified barrier during apexification procedures [2]. With a success rate of 93.33% [3]. However, despite its success rates, Ca(OH)_2_ has limitations, particularly regarding the treatment time required. On average, it takes 12.9 months for the formation of an apical calcified barrier [4], and patient compliance cannot always be guaranteed. Moreover, prolonged application of Ca(OH)_2_ increases the risk of losing the coronal seal and tooth fracture. The calcified barrier formed by Ca(OH)_2_ often contains porosities and small amounts of mineral deposits [4,5]. Mineral trioxide aggregate (MTA) has emerged as the gold standard and the preferred material to address the limitations of Ca(OH)_2_. It offers excellent sealing properties, biocompatibility, antibacterial properties, and the ability to form apical hard tissue [6]. Unlike Ca(OH)_2_, MTA does not compromise the mechanical properties of dentin. Additionally, MTA placement is a single-step procedure and does not require multiple applications over time like Ca(OH)_2_ [4].

This paper aims to describe the management of an immature central incisor with necrotic pulp and apical periodontitis using MTA apical plug, with a follow-up period of 24 months.

## Case presentation

A 19-year-old male patient was referred to the Department of Conservative Dentistry and Endodontics by his general dental practitioner with complaints of pain and swelling in the mandibular region. Clinical and radiographic examination revealed the presence of an acute apical abscess associated with localized swelling. A history of trauma to an immature central incisor presented with persistent pain and swelling. Clinical and radiographic findings confirmed an acute apical abscess in a tooth with an open apex.

Extraoral examination showed no abnormalities. The patient presented with orthodontic brackets on the affected tooth, which had been placed privately by an unqualified practitioner. Before initiating treatment, all orthodontic appliances were removed, and the patient was thoroughly motivated and instructed on proper oral hygiene practices. 

**Figure 1:**
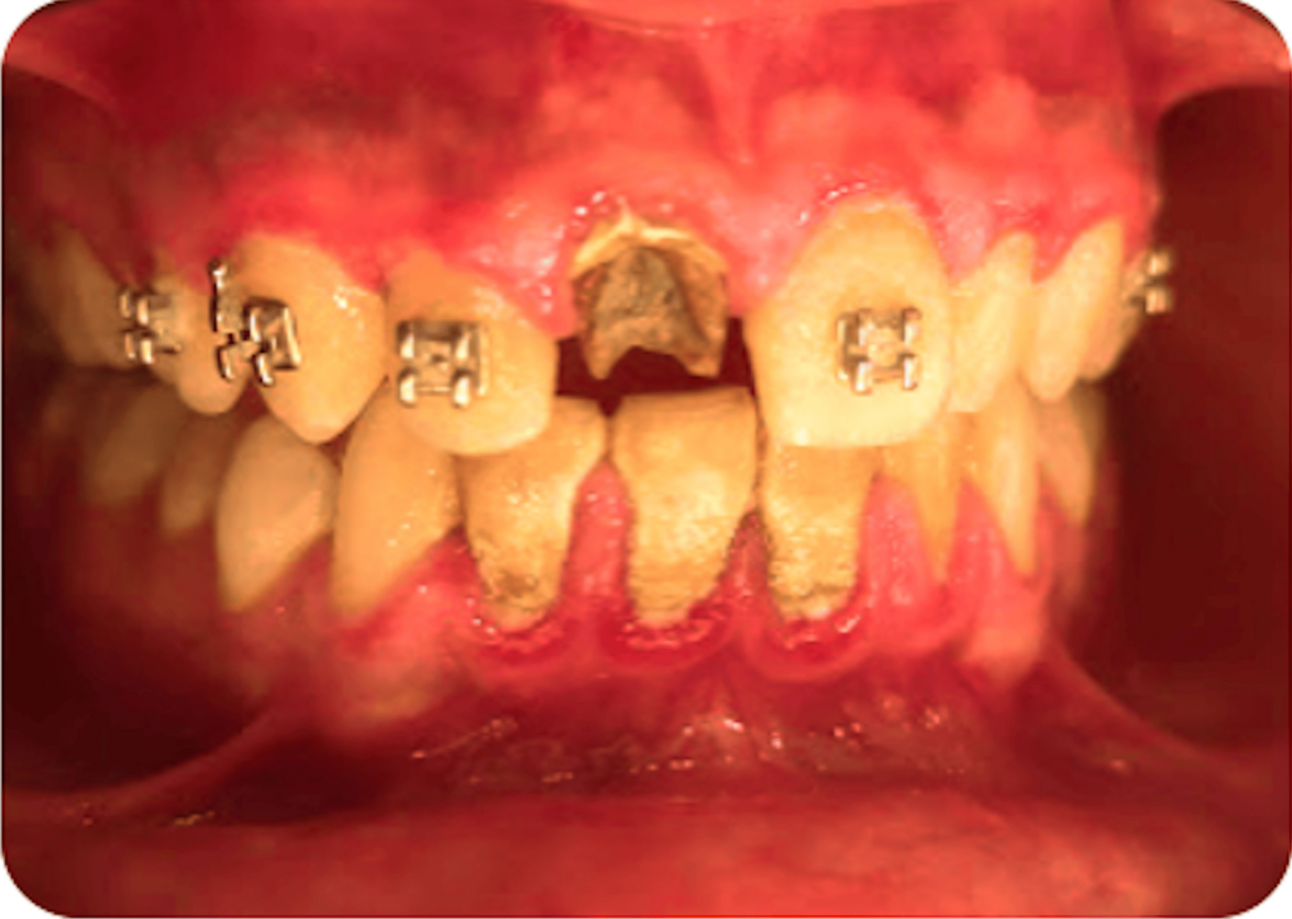
Preoperative clinical presentation showing poor oral hygiene combined with a complicated coronal fracture of the upper central incisor (#11).

Intraoral examination showed that the upper first incisor presents a complicated coronal fracture (Figure 1).

Percussion and thermal tests were negative, and the periodontal probing was within normal limits. It was noted that the tooth was grade I mobility. There was also evidence of periapical inflammation around the root apex.

**Figure 2:**
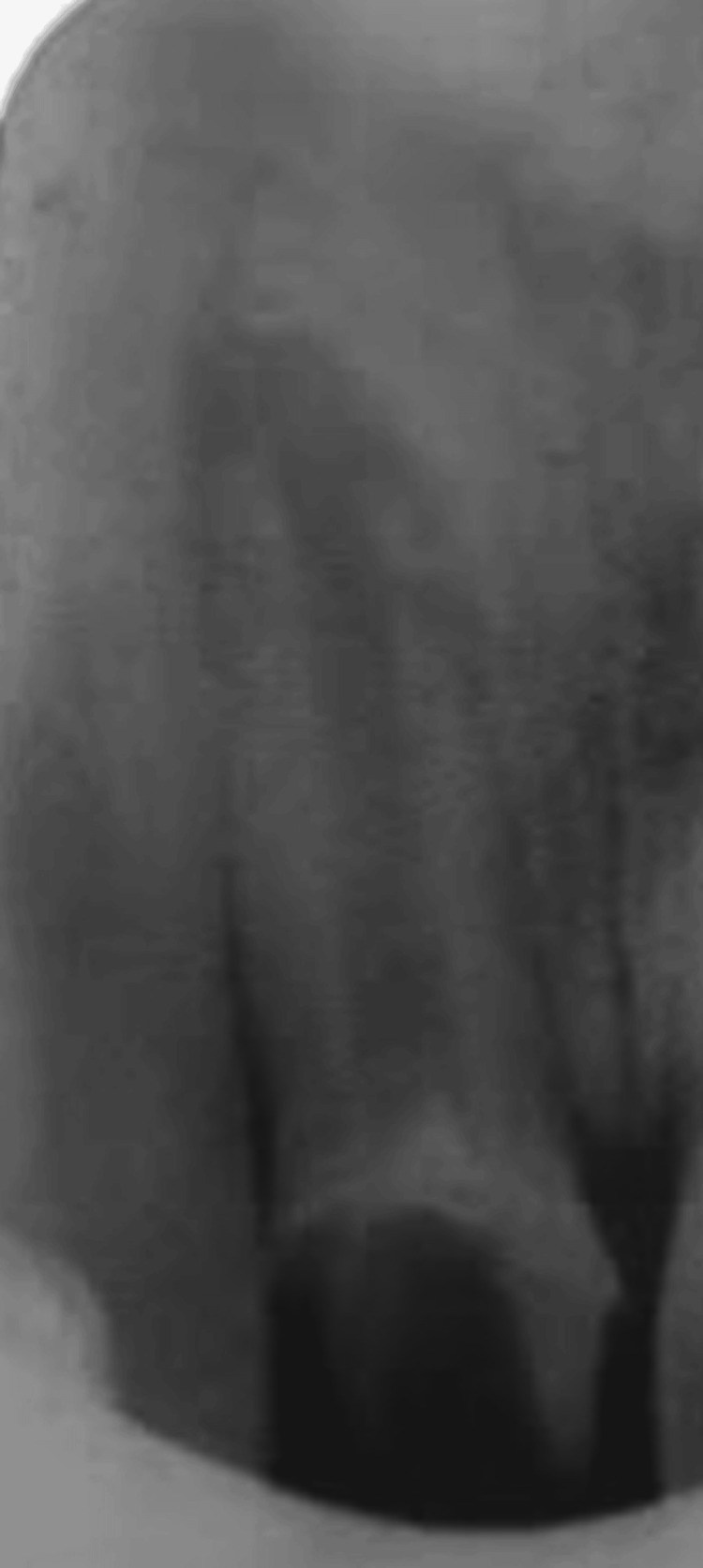
Preoperative radiograph showing an open apex, thin root walls, and a large canal space.

Radiographic examination revealed a large canal space with a wide-open apex and thin root walls (Nolla's stage 8: short root with thin walls) (Figure 2).

**Figure 3:**
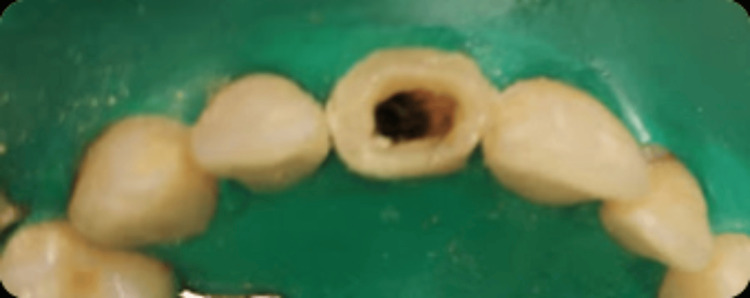
Pre-endodontic restoration and rubber dam placement.

A decision was made to create an apical barrier using MTA. Following tooth isolation, a pre-endodontic restoration was performed (Figure 3). Copious irrigation was carefully performed using (5%) sodium hypochlorite.

**Figure 4:**
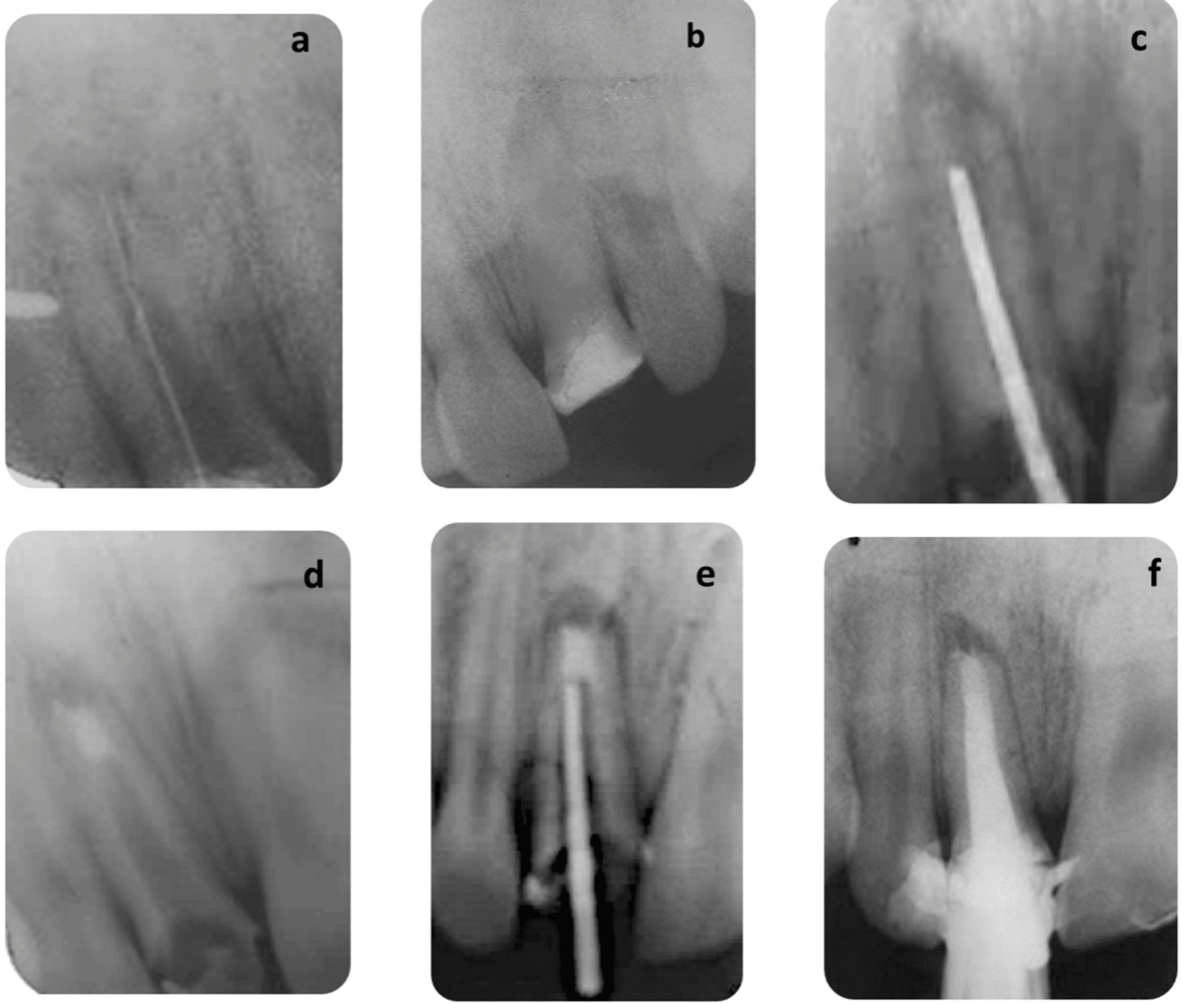
Apexification protocol: (a) working length determination; (b) placement of calcium hydroxide; (c) radiograph for plugger calibration; (d) apical plug with 3 mm of MTA; (e) radiograph showing gutta-percha cone placement; (f) periapical radiograph showing final obturation with gutta-percha. MTA, mineral trioxide aggregate

The canal was meticulously cleaned using the XP Endo Shaper® and Finisher® system with gentle instrumentation. Next, a working length (WL) radiograph was taken using a size 10 K-file (Figure 4a). It was then obturated with Ca(OH)_2_ (Figure 4b). The patient returned for a follow-up appointment two weeks later, where clinical examination showed no signs of infection. The MTA apical plug was then placed.

The Machtou plugger No. 2 was selected to match the canal diameter, with its insertion controlled to 4 mm short of the WL (Figure 4c).

The ProRoot MTA® (Dentsply Sirona, Tulsa, OK) plug was accurately positioned in the apical 4 mm of the canal, followed by radiographic verification (Figure 4d). A moist cotton pellet was then placed into the canal, and a hermetic coronal temporary restoration was applied.

After one week, the hardness of the MTA plug was controlled, and the rest of the canal space was obturated using the lateral condensation technique (Figures 4e-4f). 

After four weeks, the patient showed no signs of infection. 

**Figure 5:**
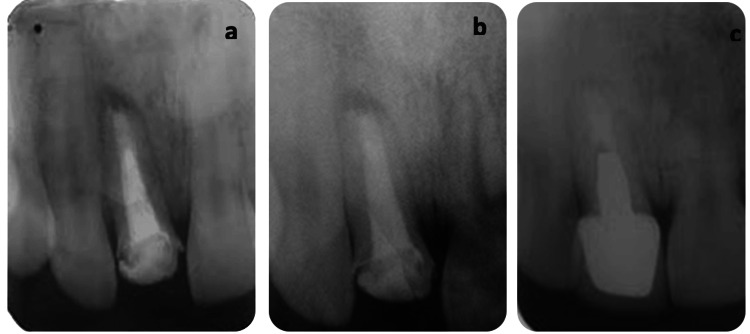
Follow-up at (a) one month, (b) three months, and (c) 24 months, showing progression with prosthetic rehabilitation.

**Figure 6:**
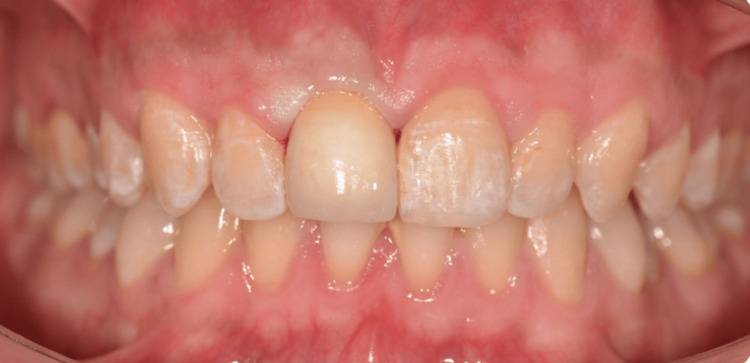
Final result following improved oral hygiene and prosthetic rehabilitation.

Then the patient was re-examined after three months (Figure 5a), 12 months (Figure 5b), and 24 months (Figure 5c), showing bone regeneration and complete resolution of the periapical radiolucency. Final results demonstrated patient satisfaction, as shown in Figure 6.

## Discussion

This case presented several challenges, mainly because of the special characteristics of the tooth and the age of the patient. First, achieving adequate sealing and disinfection of the root canal system was difficult due to the wide apex of the tooth. Additionally, the existing periradicular infection further complicated the process. Moreover, the thin root walls added to the complexity, elevating the risk of procedural errors and potential complications.

The central incisor was of considerable importance because it occupied a prominent position in the esthetic zone. Preserving both the functionality and aesthetics of the tooth was extremely important, which made the treatment decision critical. In this regard, alternative treatment options such as dental implant placement or an adhesive bridge were thoroughly deliberated. However, factors such as the age of the patient, the potential impact on adjacent teeth, and the preference to maintain the natural dentition led to the exclusion of these alternatives.

Due to the specific circumstances of the case, it was felt that a comprehensive approach to treatment was required. The decision was made to treat the root of the tooth to restore its structural integrity. Chemical-mechanical root canal preparation has been a challenge due to the complex internal anatomy of the endodontic system and a wide open apex with a significant risk of extrusion of materials, debris, and fluids. Depending on several factors, including the level of infection, the size of the apex, and the patient's tissue tolerance, the concentration of sodium hypochlorite used can vary. But higher concentrations are often necessary for effective disinfection. A higher concentration of sodium hypochlorite, such as 5.25%, may be used in open apex cases where the infection may be more severe and difficult to treat. However, it is important to ensure that the surrounding tissue is not damaged [7].

MTA-based apical plugs are a viable treatment solution in contemporary endodontic practice. This approach offers several advantages, especially for the treatment of non-vital immature permanent teeth. It serves as an alternative to traditional apexification procedures, which can be lengthy and require multiple appointments.

By employing MTA apical plugs, the treatment aimed to achieve effective sealing of the root canal system, promote periapical healing, and facilitate the continued development of root structures. This technique is consistent with the fundamental principles of biological endodontics, which prioritize maintaining tooth vitality and achieving long-term outcomes [8]. In 1993, Lee et al. first described the MTA [8].

In 1993, Lee et al. first described the MTA. MTA has been widely recognized for its exceptional properties that are contributors to its effectiveness in endodontic applications [8]. MTA is renowned for its superior sealing ability, reduced leakage rates, enhanced antibacterial properties, excellent marginal adaptation, and heightened biocompatibility [8]. MTA's ability to create a tight seal within the root canal system is unparalleled, crucial for preventing bacterial ingress and promoting periapical healing [8]. Studies consistently demonstrate lower leakage rates with MTA compared to traditional root canal filling materials, ensuring the integrity of the root canal filling over time [8]. Additionally, MTA exhibits inherent antibacterial properties, effectively eliminating bacteria within the root canal system and reducing the risk of persistent infection [8]. 

However, MTA demonstrates excellent marginal adaptation to dentin surfaces, forming a seamless interface and preventing gaps and voids that could compromise the integrity of the root canal filling [8]. Furthermore, its exceptional biocompatibility ensures minimal inflammatory response, supporting tissue healing and regeneration [8].

With a pH of 12.5 and a relatively short setting time of approximately four hours, MTA has become a cornerstone material in endodontic practice [8]. It's important to mention that the setting time of MTA can vary depending on the formulation used. instance, while some formulations such as White ProRoot® MTA (Dentsply Maillefer, Ballaigues, Switzerland) may require approximately three to four hours to set, others such as MTA Angelus (Londrina, PR, Brazil) can set in as little as 15 minutes. This variability in setting time not only streamlines treatment protocols but also reduces the risk of fracture in immature roots and crowns.

Furthermore, MTA serves as an effective scaffold for promoting the formation of hard tissue barriers [8]. As a bioactive material, MTA stimulates the production of interleukins and cytokines, thereby facilitating hard tissue formation [9]. This property is particularly advantageous in cases involving pulp necrosis and inflammatory periapical lesions, as MTA can set in a moist environment, providing an ideal substrate for tissue repair and regeneration [10].

When employing the MTA plug method, it is essential to disinfect the root canal with a Ca(OH)_2_ dressing for two weeks before MTA placement [11]. This step is crucial as chemo-mechanical preparation alone may not effectively eliminate all microorganisms. Consequently, the use of Ca(OH)_2_ dressing before MTA application helps ensure a more predictable and successful outcome [9].

To ensure successful treatment outcomes, proper placement of MTA is paramount. The use of an operating microscope is recommended to facilitate the precise placement of MTA and minimize the risk of pushing the material into apical tissues [9]. In clinical cases, MTA is typically placed on the apical portions of the canals using an attached gutta-percha cone under radiographic guidance [9].

MTA cement exhibits osteogenic activity and is well tolerated by bone tissue [12,13]. However, its high alkalinity poses a risk of tissue necrosis upon direct contact with apical tissues [14,15]. To address this concern, some studies propose a combination of the apical barrier with a lyophilized collagen sponge and MTA cement, enabling root canal obturation to be performed safely and non-invasively in a single session [16].

Moreover, comparative studies have investigated the performance of Biodentine® and MTA in terms of apical sealing, a critical aspect of successful endodontic treatment [17]. Results indicate that both materials demonstrate similar sealing capacity when used as apical plugs. Regardless of the techniques employed for apical plug formation, the sealing capacity of MTA and Biodentine remains comparable [17].

However, differences in microleakage are observed when apical plugs are formed using different techniques. For instance, when forming apical plugs with sleeve obturators, there is direct transfer of the compressed calcium silicate cement mass to the apical third. This results in minimal compaction, reducing fragmentation and deformation of the total material mass. Consequently, the material remains coherent and free of voids and gaps, thereby improving the effectiveness of the seal [17].

## Conclusions

MTA provides numerous advantages over conventional techniques like Ca(OH)_2_ apexification and pulp regeneration when used as an apical barrier in apexification procedures. In contrast to Ca(OH)_2_ apexification, MTA barrier formation is self-contained and does not need multiple visits or an external factor for development. This efficient method shortens treatment time and boosts effectiveness, making it a top pick for both clinicians and patients. In addition, MTA has great sealing abilities and is biocompatible, aiding in periapical healing and maintaining tooth vitality. In general, the MTA barrier method is a beneficial option in apexification treatment, providing reliable results and simplified treatment protocols.
